# Phytoestrogens Present in Follicular Fluid and Urine Are Positively Associated with IVF Outcomes following Single Euploid Embryo Transfer

**DOI:** 10.3390/ijms241310852

**Published:** 2023-06-29

**Authors:** Roberto Gonzalez-Martin, Andrea Palomar, Alicia Quiñonero, Nuria Pellicer, Caroline Zuckerman, Christine Whitehead, Richard T. Scott, Francisco Dominguez

**Affiliations:** 1 IVIRMA Global Research Alliance, IVI Foundation, Instituto de Investigación Sanitaria La Fe (IIS La Fe), 46026 Valencia, Spain; roberto.gonzalez@ivirma.com (R.G.-M.); andrea.palomar@ivirma.com (A.P.); alicia.quinonero@ivirma.com (A.Q.); nuria.pellicer@ivirma.com (N.P.); 2 IVIRMA Global Research Alliance, IVI-RMA New Jersey, Basking Ridge, Bernards, NJ 07920, USA; czuckerman@ivirma.com (C.Z.); cwhitehead@ivirma.com (C.W.);; 3 Sidney Kimmel College of Medicine, Thomas Jefferson University, Philadelphia, PA 19107, USA

**Keywords:** phytoestrogens, daidzein, genistein, SET, IVF outcomes

## Abstract

The impact and safety of phytoestrogens, plant-derived isoflavones with estrogenic activity predominantly present in soy, on female reproductive health and IVF outcomes continues to be hotly debated. In this prospective cohort study, 60 women attending IVI-RMA New Jersey undergoing IVF with single frozen embryo transfer (SET/FET) of good-quality euploid blastocyst after PGT-A analysis were recruited. Concentrations of two phytoestrogens (daidzein and genistein) in follicular fluid (FF) and urine (U) were measured by UPLC–MSMS, both collected on vaginal oocyte retrieval day. These measurements correlated with IVF clinical outcomes. In models adjusted for age, BMI, race/ethnicity, and smoking status, higher FF phytoestrogen concentrations were significantly associated with higher serum estradiol, enhanced probability of implantation, clinical pregnancy, and live birth. Moreover, higher urine phytoestrogen concentrations were significantly associated with improved oocyte maturation and fertilization potential and increased probability of clinical pregnancy and live birth. Finally, higher FF and urine phytoestrogen concentrations were associated with a higher probability of live birth from a given IVF cycle. Our results suggest that dietary phytoestrogens improved reproductive outcomes of women undergoing IVF treatment. However, additional prospective studies are needed to optimize the use of phytoestrogens to further enhance reproductive outcomes and/or protect against reproductive insults.

## 1. Introduction

Phytoestrogens belong to the non-steroidal polyphenol family of secondary plant metabolites and are commonly consumed by humans [1]. These substances are named after their structural similarity to endogenous estrogens (i.e., a phenolic ring with a unique hydroxyl radical attached to a carbon base) that allows them to be partial peripheral agonists of estradiol receptors and have a variable affinity for each receptor type [2,3,4]. Since different types of estrogen receptors can be found on each organ, phytoestrogens can elicit (generally weak) anti-estrogenic and/or estrogenic effects depending on their concentration, presence of endogenous sex steroids or other agonists, and the target organ [2,4]. Besides their role in endocrine signaling, phytoestrogens are also involved with antioxidant effects, cell cycle regulation, inhibition of tyrosine kinase signaling, and anti-angiogenic effects [4].

At least twenty different types of phytoestrogens have been described thus far, including isoflavones (the most abundant), lignans, coumestans, and prenylflavonoids [1,3]. Phytoestrogens are mainly consumed through legumes and, to a lesser extent, vegetables, fruits, and cereals. In particular, isoflavones are mainly found in soybeans (and their by-products), lentils, barley, sunflower seeds, cauliflower, and broccoli [3]. The most abundant isoflavones, both in dietary sources and human plasma and urine, are genistein and daidzein [3], which makes them potential phytoestrogen exposure biomarkers.

The impact and safety of phytoestrogens on female reproductive health and IVF outcomes is controversial. Some epidemiological studies suggest phytoestrogen consumption provides health benefits by protecting against certain hormone-dependent cancers (i.e., breast and prostate cancer), fibroids, menopausal symptoms, cardiovascular disease, inflammation, metabolic syndromes, and obesity [1,3]. In contrast, in vitro and in vivo studies have demonstrated the endocrine-disrupting properties of phytoestrogens in both male and female reproductive tissues. For example, phytoestrogen-rich (e.g., red clover or soy-based) diets reduced fertility in sheep [5] and captive cheetahs [6] and increased meiotic alterations in mice [7]. Further, phytoestrogens have been described as having the ability to alter the hypothalamic–pituitary–gonadal axis [8,9,10], disrupt the development of the female reproductive system [9,10], or have anti-implantation effects in animal and in vitro models after phytoestrogen exposure [11,12]. Finally, a few clinical trials have associated phytoestrogen supplementation with improved reproductive outcomes, including higher ovulation rates in women with secondary amenorrhea [13] or higher implantation and pregnancy rates in women undergoing several fertility treatments [14,15]. However, in these cases, the administered supraphysiological doses exceeded what could be obtained through dietary sources.

Other studies assessing dietary exposure to phytoestrogens showed no significant associations between urinary isoflavones and fertility among three cohorts of couples with no prior history of infertility [16,17], but these findings may be biased due to the differences in fertility potential among the participating couples. In contrast, Vanegas et al. reported that dietary soy consumption was positively associated with fertilization, clinical pregnancy, and live births in infertile patients undergoing in vitro fertilization (IVF) [18]. However, in this study, soy consumption was assessed qualitatively by means of an exposure questionnaire rather than by quantitatively detecting phytoestrogens in the participants’ biofluids (which identifies phytoestrogens unknowingly consumed through dietary sources).

The aim of this study was to elucidate the association(s) between the two main phytoestrogens (daidzein and genistein) measured in follicular fluid (FF) and urine, and reproductive outcomes of women undergoing IVF after preimplantation genetic testing for aneuploidies (PGT-A) and single frozen embryo transfer (SET/FET).

## 2. Results

### 2.1. Participants Demographic Characteristics

Participants had a median age of 33.40 years [IQR: 31.37, 36.50] and BMI of 23.87 kg/m^2^ [IQR: 21.57, 26.30], were predominantly Caucasian (71.7%), and 81.7% had never smoked (Table 1).

Regarding reproductive characteristics, participants had a median AMH concentration of 3.60 ng/mL [IQR: 2.49, 5.17]. Total follicle-stimulating hormone (FSH) and luteinizing hormone (LH) doses used for stimulation were 2100.00 IU [IQR: 1800.00, 2700.00] and 1125.00 IU [IQR: 675.00, 1443.75], respectively. The median serum E2 on the trigger day was 3750.65 pg/mL [IQR: 2622.20, 5204.62] (Table 1).

Overall, the median number of oocytes recovered was 17 [IQR: 11.00, 24.25], of which 77.47 ± 14.30% were MII. Fertilization, blastulation, and euploid rates were 81.44 ± 16.29%, 55.62 ± 21.47%, and 60.17 ± 23.72%, respectively. For the 55/60 (91.7%) women who had a FET, the implantation, clinical pregnancy, and live birth rates were 80.0%, 69.1%, and 63.6%, respectively. Of the 60 women who began IVF treatment, 58.3% achieved live birth (reproductive goal) (Table 1).

No differences in demographic variables, such as age, BMI, race/ethnicity, educational level, or smoking, were observed when comparing between tertiles of daidzein, genistein, or the sum of both phytoestrogens in FF or creatinine-corrected urine (Supplemental Table S1). Likewise, a positive relationship was observed between the tertiles of daidzein, genistein, and the sum of both phytoestrogens in FF, between the concentration of serum E2 on the trigger day, and between genistein and the sum of both phytoestrogens in FF with reproductive success (Supplemental Table S1).

### 2.2. Phytoestrogen Distribution in Biofluids

The distributions and percentages above the limit of detection (LOD) of phytoestrogens in FF and urine are presented in Table 2. Daidzein and genistein were detected and quantified in 100% of the urine samples but only 53.6% and 78.6% in FF samples, respectively. The geometric means (standard deviation) of daidzein, genistein, and the sum of both phytoestrogens for the 60 samples were 0.241 (SD: 2.96), 1.195 (SD: 9.113), and 1.514 (SD: 11.44) ng/mL in FF, respectively, or 54.69 (SD: 565.26), 44.57 (SD: 281.26), and 120.41 (SD: 728.19) µg/g creatine in urine (Table 2).

Positive correlations were observed between the analyzed biofluids and each phytoestrogen. Specifically, a moderate–high correlation was observed between the phytoestrogens measured in FF and urine (r = 0.83, r = 0.8, and r = 0.72 for daidzein, genistein, and the sum of phytoestrogens, respectively) (Supplemental Figure S1). Further, for each biofluid, moderate–high positive correlations were observed between the concentrations of each phytoestrogen and their sum (Supplemental Figure S1).

### 2.3. Association of Phytoestrogen Concentrations with Ovarian Reserve, Ovarian Response, and Preimplantation IVF Outcomes

In models adjusted for age, BMI, race/ethnicity, and smoking status, higher urinary genistein concentrations were significantly associated with higher AMH (p20 vs. p80 (95% CI): 3.28 (1.06, 10.20), *p* = 0.04) and serum E2 on the day of hCG trigger (p20 vs. p80 (95% CI): 1.29 (1.02, 1.63), *p* = 0.04) (Table 3). Serum E2 was also significantly associated with higher concentration of daidzein, genistein, and the sum of both phytoestrogens in the FF (p20 vs. p80 (95% CI): 1.15 (1.00, 1.31), *p* = 0.044; 1.41 (1.09, 1.82), *p* = 0.010; and 1.33 (1.07, 1.66), *p* = 0.012, respectively) (Table 3).

Further, a significantly higher proportion of mature oocytes was associated with higher daidzein concentration in both FF and urine (p20 vs. p80 (95% CI): 1.16 (1.03, 1.31), *p* = 0.017; and 1.23 (1.06, 1.43), *p* = 0.01, respectively), in addition to the sum of phytoestrogen in urine (p20 vs. p80 (95% CI): 1.30 (1.08, 1.56), *p* = 0.01) and genistein concentration in urine (p20 vs. p80 (95% CI): 1.31 (1.03, 1.66), *p* = 0.03) (Table 3).

Data were adjusted for age (continuous), BMI (continuous), race/ethnicity, and smoking status (never, ever). Data are presented as an increase between the 20th and 80th percentiles, which, respectively, were: 0.10 and 0.34 ng/mL for daidzein, 0.20 and 3.71 ng/mL for genistein, and 0.40 and 4.30 ng/mL for the sum of both phytoestrogens detected in the follicular fluid; or 21 and 145 µg/g CR for daidzein, 10 and 149 µg/g CR for genistein, and 34 and 297 µg/g CR for the sum of both phytoestrogens detected in (creatine-corrected) urine.

Finally, a higher proportion of fertilized embryos was significantly associated with daidzein concentration in both FF and urine (p20 vs. p80 (95% CI): 1.19 (1.05, 1.35), *p* = 0.009; and 1.27 (1.09, 1.48), *p* = 0.003, respectively), and concentration of genistein and the sum of both phytoestrogens in urine (p20 vs. p80 (95% CI): 1.34 (1.05, 1.72), *p* = 0.02; and 1.34 (1.10–1.62), *p* = 0.004], respectively) (Table 3). We found no significant association between either or both phytoestrogens (detected in either biofluid) and blastulation or euploidy relative proportions (Table 3).

### 2.4. Association of Phytoestrogen Concentrations with Clinical IVF Outcomes

**Figure 1:**
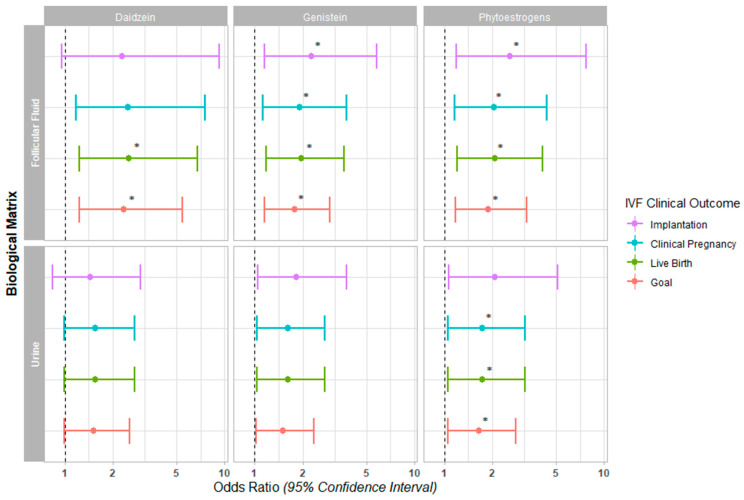
Forest plot for IVF clinical outcomes. The Odds Ratios (95% Confidence Interval) are presented for implantation, clinical pregnancy, and live birth following single frozen embryo transfer, in addition to the live birth achieved in a given IVF cycle (goal) across daidzein, genistein, and the sum of both phytoestrogens quantified in follicular fluid and urine (* *p* < 0.05).

The associations between the concentrations of daidzein, genistein, and both phytoestrogens in FF and urine with IVF outcomes (i.e., implantation, clinical pregnancy, and live birth per SET/FET) or the reproductive goal (live newborn per IVF treatment initiated) were evaluated (Figure 1, Supplemental Table S2).

In our fully adjusted models, a higher concentration of both phytoestrogens in FF was associated with a significantly increased probability of implantation (OR (95% CI): 2.57 (1.19, 7.74), *p* = 0. 045), clinical pregnancy (OR (95% CI): 2.05 (1.16, 4.38), *p* = 0.031), live birth (OR (95% CI): 2.07 (1.21, 4.12), *p* = 0.018), and achieving the reproductive goal (OR (95% CI): 1.88 (1.18, 3.29), *p* = 0.015) (Figure 1, Supplemental Table S2). When individually evaluating each metabolite in the FF, genistein was significantly associated with the probability of implantation (OR (95% CI): 2.27 (1.15, 5.8), *p* = 0.043) and clinical pregnancy (OR (95% CI): 1.91 (1.13, 3.75), *p* = 0.030). Daidzein and genistein were both significantly associated with the probability of achieving live birth (OR (95% CI): 2.51 (1.24, 6.76), *p* = 0.031; and 1.95 (1.18, 3.63), *p* = 0.018, respectively) and the reproductive goal (OR (95% CI): 2.33 (1.23, 5.44), *p* = 0.024; and 1.78 (1.16, 2.97), *p* = 0.015, respectively) (Figure 1, Supplemental Table S2).

In the case of urine, both phytoestrogens together were associated with a significantly increased probability of achieving a clinical pregnancy (OR (95% CI): 1.73 (1.06, 3.18), *p* = 0.047), live birth (OR (95% CI): 1.73 (1.06, 3.18), *p* = 0.047), and the reproductive goal (OR (95% CI): 1.65 (1.05, 2.8), *p* = 0.043); however, when considered individually, they were not associated with improved clinical IVF outcomes in the fully adjusted models (Figure 1, Supplemental Table S2).

## 3. Discussion

We compared the IVF outcomes of 60 women who underwent PGT-A for SET/FET with phytoestrogen concentrations in their FF and urine on the day of VOR. In our study population, higher phytoestrogen concentrations had significant positive associations with ovarian response to stimulation (i.e., E2 production and oocyte maturation) and preimplantation IVF outcomes (i.e., fertilization potential). In addition, in our study population, the sum of phytoestrogens detected in the biofluids significantly favored clinical IVF outcomes following SET/FET (i.e., implantation, clinical pregnancy, live birth) and the probability of achieving a live birth in a given IVF cycle.

To our knowledge, this is the first study directly evaluating the presence of phytoestrogens in biofluids with IVF outcomes of women undergoing SET/FET. Urinary daidzein and genistein concentrations found in our population were similar to those described for the general U.S. population [19]. Our findings agree with a previous observational study of 315 subfertile American women who collectively underwent 530 assisted reproduction cycles, which reported a significant positive correlation between dietary soybean consumption and fertilization, pregnancy, and live birth rates [18]. Further, a randomized clinical study by Shahin et al. [15] evaluated the impact of phytoestrogen supplementation (120 mg/day during the first 12 cycle days) in 147 women with unexplained infertility having timed intercourse. The women who received phytoestrogen supplementation in addition to clomiphene citrate for ovarian stimulation had significantly improved clinical pregnancy rates than those that did not [15]. Similarly, a randomized clinical trial by Unifer et al. with 213 women collectively undergoing 284 IVF cycles found a significant increase in implantation, clinical pregnancy, and ongoing pregnancy/delivered rates among women who received luteal phase support with a daily isoflavone supplement (1500 mg/day), rather than a placebo [14].

Our findings, taken together with the results of these two clinical trials evaluating the potential benefits of phytoestrogen supplementation, suggest that isoflavones may act through mechanisms that modulate endometrial response to estrogen. Although, both studies observed an increase in endometrial thickness after phytoestrogen supplementation [14,15], this may have been a confounding effect of the clomiphene citrate having a deleterious impact on endometrial function [20]. Indeed, the study by Vanegas et al. did not associate this increased endometrial thickness to women who self-reported higher dietary soy consumption [18]. With our study design, the potential confounding effect of ovarian hyperstimulation on endometrial receptivity was avoided by delaying SET/FET. In addition, the transfer of a single euploid embryo suppressed bias(es) due to possible implantation failures of embryonic origin, allowing us to reliably assess endometrial function. The findings of our study suggest that the presence of phytoestrogens increases embryo implantation and facilitates pregnancy maintenance, although we cannot say whether this is due to their effect on the endometrium, the embryo, or a combination of both. More detailed mechanistic studies would be necessary for an in-depth study of this association.

Based on the premise that improved oocyte competence may be partly responsible for the clinical results found by Vanegas et al. [18], we postulated that in our cohort of women, those with increased phytoestrogen concentrations on the day of VOR had a better ovarian response, which improved oocyte maturation and fertilization rates. Indeed, this theory of the positive effects of phytoestrogens on ovarian function was reinforced by a clinical trial where improvements in ovulation and E2 production were observed in anovulatory women with daily supplementation of 6 g black soybean powder [13].

Finally, phytoestrogens may protect female reproductive function. In addition to the benefits of supplementation on the endometrium of women treated with clomiphene citrate we discussed above, an observational study in humans and preclinical experiments in rodents have shown soy-rich diets counteract endocrine-disrupting substances such as bisphenol A [7,21]. Furthermore, phytoestrogens have also been reported to protect against iatrogenic infertility caused by pelvic irradiation [22]. This indicates other potential mechanisms by which phytoestrogens could improve IVF treatment outcomes.

Our study presents a series of limitations that restrict the interpretation of the results presented. First, our findings are relevant and applicable to subfertile women whose reproductive outcomes are most susceptible to the impacts of environmental factors [23]. Other design limitations are that paternal exposure to phytoestrogens was not taken into account, and the results should be contextualized to the geographic reality and lifestyle of the population studied (different results may be observed in other populations). In addition, we only evaluated the presence of phytoestrogens in biofluids obtained on VOR day, so evaluation at other times may be more informative. Additionally, we only evaluated the presence of the two most abundant phytoestrogens. Thus, further research is required to validate if other phytoestrogens have similar implications in human female reproduction. Finally, the findings from this study should be interpreted with caution, as causality in the observed relationships cannot be assured.

The strengths of our study include its prospective design, which minimized the possibility of reverse causation. We diligently selected a cohort of patients that was subjected to robust standard operating procedures in the laboratory to reduce biases in the assisted reproduction techniques and critically evaluate the reproductive outcomes. Performing PGT-A ensured the transfer of a euploid embryo while performing a delayed SET/FET eliminated the confounding effect of ovarian stimulation on endometrial function. Another strength was the evaluation of metabolites in biofluids that are routinely collected for VOR and reflect the ovarian microenvironment (in the case of the FF). Overall, our model reliably assessed the impact of environmental factors on IVF clinical outcomes, and we propose FF as the most relevant biofluid for predicting the reproductive outcomes of IVF patients.

## 4. Materials and Methods

### 4.1. Study Population

Sixty women (aged 18–42 with a body mass index (BMI) between 18.6–29.9) undergoing IVF treatment with antagonist ovarian stimulation cycles, PGT-A, and SET/FET between 2018 and 2019 at RMANJ—Basking Ridge were included in the study. Patients were excluded from the study if they had severe male factor infertility, a known history of endometrial insufficiency (i.e., abnormal or unrepaired uterus, irregular [or <7 mm thick] endometrium on the day of SET/FET), altered karyotypes, thrombophilia, or uncorrected systemic or endocrine pathologies.

### 4.2. Collection of Follicular Fluid and Urine Samples

Urine was collected from fasting participants on the morning of vaginal oocyte retrieval (VOR) in sterile polypropylene containers and kept at 4 °C. Samples were centrifuged at 500× *g* for 7 min to pellet the sediment, and the supernatant (urine) was aliquoted and stored at −80 °C. Follicular fluid was then aspirated during VOR. After isolation of the cumulus–oocyte complexes, each participant’s pooled FF was centrifuged at 1000× *g* for 3 min to remove cellular debris, aliquoted, and stored at −80 °C. Once all the study samples were obtained, they were sent to IVI Foundation (Valencia, Spain) on dry ice and transferred to the analytical unit of the IIS La Fe (Valencia, Spain) for daidzein and genistein quantification.

### 4.3. Phytoestrogen Quantification by UPLC–MS/MS

The concentrations of total daidzein and genistein (free + conjugated) in FF and urine were measured by ultra-performance liquid chromatography coupled with tandem mass spectrometry (UPLC–MS/MS). First, 600 μL of each sample was subjected to enzymatic hydrolysis of glucuronidase conjugates, employing β-glucuronidase from *E. coli* K12. Subsequently, a liquid–liquid extraction was performed by adding 600 μL of ethyl acetate, vigorously agitating for 30 s, and mixing for 15 min with a sample rotator. This extraction process was repeated, and the resulting two ethyl/acetate fractions were combined, dried in a SpeedVac concentrator, and stored at −80 °C.

All samples were analyzed using a UPLC–MS/MS, Triple Quad 1290-6460 (Agilent, Santa Clara, CA, USA) through positive electrospray ionization (ESI+). Thawed residues were reconstituted in 60 μL of water:acetonitrile (70:30, *v*/*v*). The water and acetonitrile, both containing 0.1% formic acid, were used as mobile phases. The limit of detection (LOD) was set to 0.5 ng/mL for both daidzein and genistein. In addition to the study samples, each analytical run included low- and high-concentration quality control materials and blank reagents to ensure data accuracy and reliability.

The creatinine concentration was used to correct phytoestrogen concentrations for urine dilution. Creatinine concentration was analyzed by the Jaffe reaction (where creatinine and alkaline picric acid produces a red/orange complex) using a commercial kit (KGE005, R&D Systems, Minneapolis, MN, USA) in aliquoted urine samples stored at −80 °C. The nanograms of metabolite per gram of creatinine was calculated, and phytoestrogen concentrations below the LOD were assigned a value equal to half of the LOD.

### 4.4. Clinical Management and Outcome Assessment

Patient baseline characteristics (i.e., date of birth, weight, and height) were collected at enrollment. The BMI was calculated as the ratio of weight (kilograms) to height (meters) squared. Other demographic variables, such as race/ethnicity, education, or smoking, were self-reported on a questionnaire. The patients’ most recent anti-müllerian hormone (AMH) levels and reproductive outcomes were retrieved from their electronic medical records, while serum estradiol (E2) concentration was measured in blood collected on the trigger day, using an in-clinic automated electrochemiluminescence immunoassay.

Controlled ovarian stimulation was conducted in all patients using a gonadotropin-releasing hormone (GnRH)-antagonist. Following standard clinical protocol, gonadotrophin doses were individualized based on the clinician’s discretion and estimation of the participant’s ovarian reserve. Final oocyte maturation was induced with human chorionic gonadotropin (hCG) and/or a GnRH-agonist trigger when the bulk of recruited follicles reached a 15–20 mm diameter, and VOR was performed by ultrasound-guided aspiration 36 h later [24,25].

Oocyte yield and maturation rate was recorded following stripping of the cumulus–oocyte complexes. Intracytoplasmic sperm injection (ICSI) was performed in all cases, regardless of sperm parameters, to reduce the possibility of DNA contamination during PGT-A and standardize the fertilization method. Oocyte fertilization was evaluated approximately 18 h after ICSI, and embryos were cultured in vitro until the blastocyst stage using sequential culture medium. Subsequently, a trophectoderm biopsy was performed for PGT-A, and the embryos were vitrified [24,25].

The endometrial priming protocols consisted of a first phase of oral estrogens followed by intramuscular progesterone. On the day of transfer, a single euploid embryo was thawed and transferred into the endometrial cavity using an ultrasound-guided catheter.

The clinical IVF outcomes were evaluated according to the clinic’s protocols. Successful implantation was defined as a serum β-hCG level > 5 mIU/mL, assessed 9 days after FET. Clinical pregnancy was confirmed by ultrasound visualization of an intrauterine sac with viable fetus 6 weeks after FET. A live birth was defined as the birth of a neonate after at least 24 weeks of gestation [24,25].

### 4.5. Statistical Analysis

Participant baseline demographics and reproductive characteristics were presented as median ± interquartile ranges (IQR) or percentages. Associations between the phytoestrogen concentrations and the baseline demographic and reproductive characteristics were evaluated using Kruskal–Wallis tests for continuous variables and chi-square tests for categorical variables. Spearman correlation matrices were generated to examine the relationships between the biofluids for each of the phytoestrogens and the different phytoestrogens in each biofluid.

Generalized linear multivariate mixed models with random intercepts were employed to evaluate the association between phytoestrogen concentrations (i.e., of daidzein, genistein, or the sum of both phytoestrogens) in FF and urine samples and IVF outcomes. Mean differences for AMH and E2 on the trigger day were estimated by specifying a Gaussian distribution and an identity link function. Meanwhile, for the total number of retrieved oocytes, and relative proportions of mature oocytes, fertilized embryos, blastocysts, and euploid embryos, a Poisson distribution with a log link function for discrete count variables was employed. The oocyte yield, number of mature oocytes, blastocysts, and embryos evaluated were respectively applied as offsets for the relative proportions. Phytoestrogen concentrations were modeled as continuous (log-transformed), and linear associations were obtained by comparing the increase between the 20th and 80th percentile. Finally, a binomial distribution and a logit link function were used to calculate the odds ratios for clinical outcomes (i.e., implantation, clinical pregnancy, and live birth) relative to the FET and reproductive goal (i.e., probability of a live birth for a given cycle).

For a better interpretation of the results, the marginal population means adjusted for all the covariates of the model are presented. Variables measured as potential confounders included factors previously related to IVF outcomes. Final models were adjusted for age (continuous), BMI (continuous), race/ethnicity, and smoking status (i.e., never, ever). In all cases, *p* < 0.05 was considered statistically significant.

All statistical analyses were performed using R software (version 3.6.2). The “tableone” package was used to calculate overall participants’ demographic, reproductive characteristics, and differences among phytoestrogen tertiles [26]; the “corrplot” package was used to generate the correlation matrices [27]; the “questionr” package was employed to estimate the odds ratios from the binomial model results [28].

## 5. Conclusions

In conclusion, women undergoing PGT-A SET/FET IVF treatment with higher phytoestrogen concentrations in follicular fluid and urine on the day VOR showed a significant increase in estradiol levels on the trigger day, more mature oocytes, more fertilized embryos, and better clinical IVF outcomes, which ultimately increased their likelihood of giving birth to a live newborn. Our findings are consistent with those reported in other studies, suggesting a beneficial effect of phytoestrogen exposure in women undergoing IVF treatment. Additional prospective studies are needed to optimize the use of phytoestrogens for further improving reproductive outcomes and/or protecting against reproductive insults.

## Figures and Tables

**Table 1:** Baseline demographic and reproductive participant characteristics.

**Demographic characteristics**	
Age (years), median [IQR]	33.40 [31.37–36.50]
Body mass index (kg/m^2^), median [IQR]	23.87 [21.57–26.30]
Race/ethnic group, *n* (%)	
White/Caucasian	43 (71.7%)
Afro-American	2 (3.3%)
Asian	6 (10.0%)
Hispanic	6 (10.0%)
Other	3 (5.0%)
Education level, *n* (%)	
>High school	55 (94.8%)
Smoking habit, *n* (%)	
Never Smoked	49 (81.7%)
Ex-smoker	10 (16.7%)
Passive smoker	1 (1.7%)
**Reproductive characteristics**	
Serum AMH (ng/mL), median [IQR]	3.60 [2.49–5.17]
Initial GnRH Antagonist stimulation protocol, *n* (%)	60 (100.0%)
Total FSH dose during stimulation (IU), median [IQR]	2100.00 [1800.00–2700.00]
Total LH dose during stimulation (IU), median [IQR]	1125.00 [675.00–1443.75]
Serum E2 on trigger day (pg/mL), median [IQR]	3750.65 [2622.20–5204.62]
Number of retrieved oocytes, median [IQR]	17.00 [11.00–24.25]
Oocyte maturation rate, % mean ± SD	77.47 ± 14.30%
Fertilization rate, % mean ± SD	81.44 ± 16.29%
Blastulation rate, % mean ± SD	55.62 ± 21.47%
Euploid rate, % mean ± SD	60.17 ± 23.72%
Transfer rate, *n* (%)	55 (91.7%)
Implantation (positive hCG) rate, *n* (%)	44 (80.0%)
Clinical pregnancy rate, *n* (%)	38 (69.1%)
Live newborn rate, *n* (%)	35 (63.6%)
Reproductive goal rate, *n* (%)	35 (58.3%)

Abbreviations: AMH, anti-müllerian hormone; E2, estradiol; FSH, follicle-stimulating hormone; hCG, human chorionic gonadotropin; LH, luteinizing hormone.

**Table 2:** Distribution of phytoestrogen concentrations among follicular fluid and urine samples collected on the day of vaginal oocyte retrieval.

	LOD	Detected (%)	GM ± SD	Minimun	25%	50%	75%	Maximun
Follicular Fluid								
Daidzein (ng/mL)	0.25	53.60%	0.24 (2.96)	0.10	0.10	0.17	0.32	20.94
Genistein (ng/mL)	0.25	78.60%	1.19 (9.11)	0.20	0.47	0.81	2.78	44.74
∑ phytoestrogens (ng/mL)	N/A	N/A	1.51 (11.44)	0.30	0.60	1.10	3.44	57.88
Urine								
Daidzein (ng/mL)	0.25	100%	47.64 (685.16)	3.45	16.24	36.56	137.69	3725.67
Creatinine Corrected (µg/g CR)			54.69 (565.26)	3.74	7.84	23.64	47.32	124.80
Genistein (ng/mL)	0.25	100%	38.83 (348.60)	0.81	10.91	43.79	101.73	2010.60
Creatinine Corrected (µg/g CR)			44.57 (281.26)	2.76	7.04	16.10	35.45	114.57
∑ phytoestrogens (ng/mL)	N/A	N/A	104.88 (916.19)	4.69	39.65	77.76	243.17	4395.50
Creatinine Corrected (µg/g CR)			120.41 (728.19)	9.71	42.37	100.90	253.55	3988.65

Abbreviations: CR, Creatinine; GM, Geometric Mean; LOD, Limit of Detection; N/A, Not applicable; SD, Standard Deviation.

**Table 3:** Mean differences in ovarian response and preimplantation IVF outcomes by phytoestrogen concentration.

	Anti-Mullerian Hormone		Serum Estradiol on Day of hCG Trigger		Number of Retrieved Oocytes		Mature Oocytes		Fertilized Embryos		Blastocysts		Euploid Embryos	
	p20 vs. p80 (95% CI)	*p*	p20 vs. p80 (95% CI)	*p*	p20 vs. p80 (95% CI)	*p*	p20 vs. p80 (95% CI)	*p*	p20 vs. p80 (95% CI)	*p*	p20 vs. p80 (95%CI)	*p*	p20 vs. p80 (95%CI)	*p*
Follicular Fluid														
Daidzein	1.43 (0.75–2.76)	0.273	1.15 (1.00–1.31)	0.044	1.10 (0.95–1.27)	0.195	1.16 (1.03–1.31)	0.017	1.19 (1.05–1.35)	0.009	1.11 (0.97–1.27)	0.134	1.04 (0.89–1.21)	0.604
Genistein	3.00 (0.84–10.74)	0.089	1.41 (1.09–1.82)	0.010	1.13 (0.82–1.56)	0.456	1.23 (0.92–1.65)	0.166	1.28 (0.94–1.75)	0.112	1.12 (0.82–1.52)	0.481	1.00 (0.71–1.40)	0.997
Phytoestrogens	2.51 (0.84–7.46)	0.097	1.33 (1.07–1.66)	0.012	1.13 (0.86–1.48)	0.382	1.22 (0.96–1.56)	0.105	1.27 (0.98–1.64)	0.066	1.12 (0.86–1.45)	0.388	1.02 (0.77–1.35)	0.903
Urine														
Daidzein	1.45 (0.61–3.44)	0.389	1.13 (0.95–1.35)	0.176	1.10 (0.91–1.34)	0.300	1.23 (1.06–1.43)	0.007	1.27 (1.09–1.48)	0.003	1.12 (0.95–1.32)	0.171	1.07 (0.89–1.28)	0.450
Genistein	3.28 (1.06–10.20)	0.040	1.29 (1.02–1.63)	0.036	1.21 (0.92–1.59)	0.175	1.31 (1.03–1.66)	0.030	1.34 (1.05–1.72)	0.020	1.21 (0.94–1.56)	0.133	1.14 (0.86–1.51)	0.352
Phytoestrogens	2.34 (0.84–6.52)	0.103	1.23 (1.00–1.52)	0.054	1.17 (0.93–1.47)	0.188	1.30 (1.08–1.56)	0.007	1.34 (1.10–1.62)	0.004	1.17 (0.95–1.43)	0.133	1.12 (0.89–1.40)	0.320

## Data Availability

The data presented in this study are openly available in Mendelei.

## Supplementary Materials

The following supporting information can be downloaded at: https://www.mdpi.com/article/10.3390/ijms241310852/s1.
