# Supplementary material for: Phytoestrogens Present in Follicular Fluid and Urine Are Positively Associated with IVF Outcomes following Single Euploid Embryo Transfer
Source: Int J Mol Sci. 2023 Jun 29;24(13):10852. doi: 10.3390/ijms241310852 (PMC10342078; doi:10.3390/ijms241310852)
Supplement: Supplementary file 1 [file ijms-24-10852-s001.zip › ijms-2449012-Supplementary.pdf]

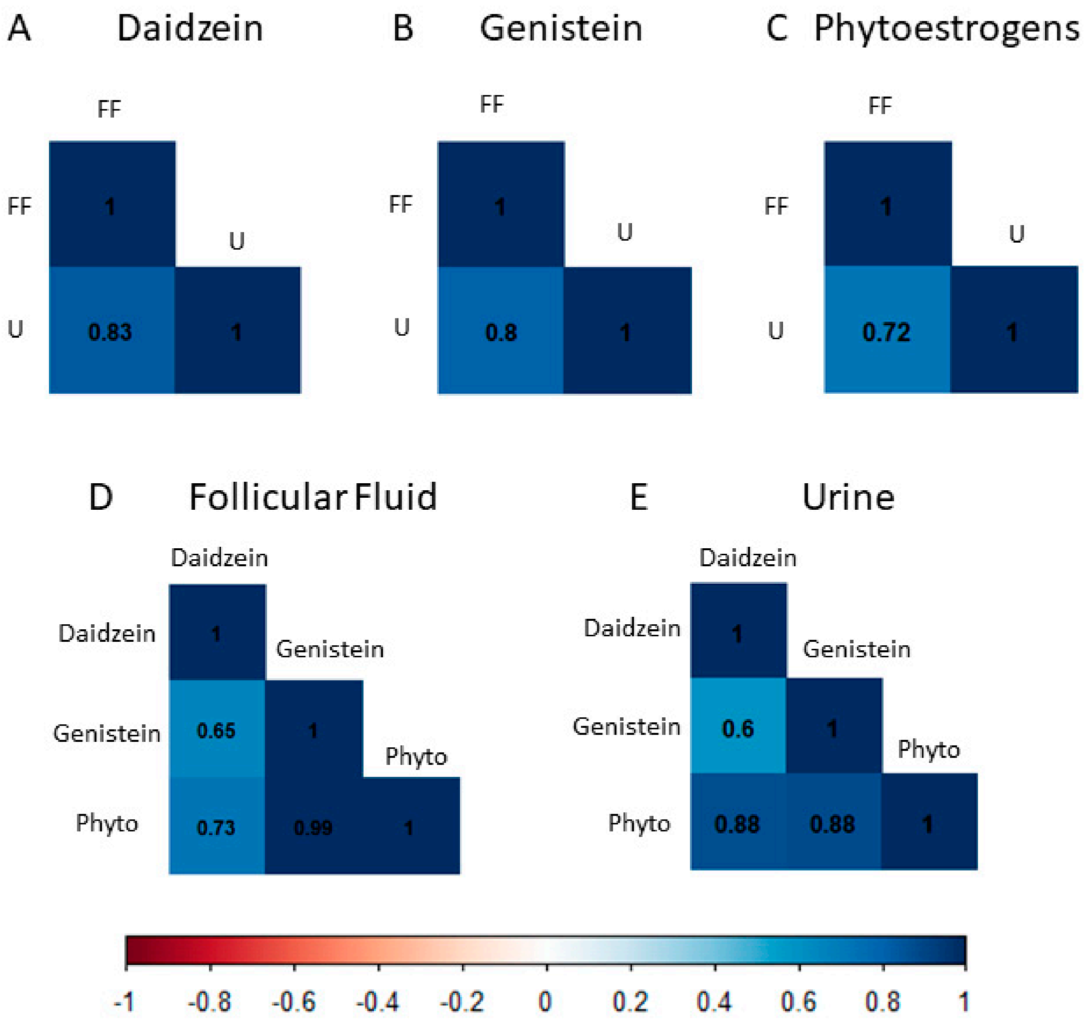

Figure S1. Spearman correlation matrix representing the relationships between phytoestrogens and biofluids.
